# Distribution of Number, Location of Pain and Comorbidities, and Determinants of Work Limitations among Firefighters

**DOI:** 10.1155/2020/1942513

**Published:** 2020-11-08

**Authors:** Goris Nazari, Temitope A. Osifeso, Joy C. MacDermid

**Affiliations:** ^1^School of Physical Therapy, Faculty of Health Science, Western University, London, ON, Canada; ^2^Collaborative Program in Musculoskeletal Health Research, Bone and Joint Institute, Western University, London, ON, Canada; ^3^School of Rehabilitation Science, McMaster University, Hamilton, ON, Canada; ^4^Roth McFarlane Hand and Upper Limb Centre, St. Joseph's Hospital, London, ON, Canada

## Abstract

**Introduction:**

The unique demands of firefighting results in acute, recurrent, or chronic pain complications. We aimed to describe the percentage distribution of number and location of painful sites among FFs and determine whether work limitations differed based on the number or location of painful sites, age, and/or sex.

**Methods:**

About 325 firefighters completed a work limitation questionnaire (WLQ-26) and a checklist to indicate painful regions of the body using either a paper format or an online survey. A one-way ANOVA was employed to analyze the transformed work limitation scores; this was a two-sided test with a significance level of <0.05, to determine if work limitations differed among firefighters based on the number or location of painful sites, age, and/or sex.

**Results:**

The data analyzed consisted of 325 (men = 216, women = 109) FFs in total. The percentage distribution of the number of painful sites in our study cohort was 43% no pain, 17% one painful site, 19% two painful sites, and 21% three or more painful sites. The percentage distribution of the locations of painful sites was 43% no pain, 41% spine, 9% lower extremity, and 7% upper extremity. An estimated 31% of FFs (*n* = 102) reported non-MSK comorbidities with 23% (*n* = 76) reporting at least one non-MSK comorbidity and 8% (*n* = 26) reported having two or more comorbidities. FFs > 45 years of age experienced more physical work limitations than FFs ≤ 45years (mean difference: 0.74/10; 95% CI .19-1.29; *p* = 0.008).

**Conclusions:**

The majority of firefighters reported having at least one painful site and indicated the spine as the most common painful location. Age, the number of painful sites, and location of pain were identified as a potential contributor to physical/mental and work output limitations.

## 1. Background

Firefighting is widely recognized as an inherently dangerous occupation [1–4]. It has one of the highest prevalence of occupational injuries and fatality rates when compared to other working populations [5]. Firefighters (FFs) have high rates of work-related pain [6], and they are three times more predisposed to injuries than workers in the private sector [7]. This may require adopting awkward or restricted postures, lifting heavy loads, or sustained work over long periods of time [1–4, 8]. Such repetitive trauma or overexertion injuries predispose FFs to sprains and strains that often result in work limitations [9]. Work limitations are defined as the degree to which health challenges influence specific aspects of job performance [10].

In 2016, despite the decrease in the total number of firefighter injuries reported by the National Fire Protection Association in the United States, firefighter injuries remained high—62,085 injuries [3, 11]. Strain, sprain, and muscular pain constituted for 45.7% of all injuries received during the fireground operations (at-work injuries) [11, 12]. Although FFs often report work limitations, there is little or no evidence that the number or location of painful sites impact work limitations. In addition, women in the fire service occupy a small percentage of FFs in North America; hence, relatively little is known about their work limitations or occupational health concerns as well. Furthermore, there is a major economic burden and cost associated with occupational injuries among FFs as the cost of pain secondary to occupational injuries sustained annually is about $900 million with an average cost of about 5000 USD per person [6]. Approximately a third of claims made by FFs are related to work-related pain, and more than 80% is associated with sprains and strains [6, 13].

Despite the evidence of prevalence, lost time at work, and cost of work-related pain in FFs, there is a paucity of reports concerning the impact of at-work limitations among FFs. There has also been a growing concern on the prevalence of mental health challenges including posttraumatic stress disorder among FFs and the impact on their work ability in fire service [14, 15]. For example, the economic cost of depression in Canada ranged from $14.4 billion to $33 billion for health expenses and at-work disability costs (workers' compensation claims) [16]. Studies investigating the relationship between nonmusculoskeletal (MSK) comorbidity and work limitations in FFs are sparse.

### 1.1. Study Objectives

Therefore, the aims of this study were (1) to describe the percentage distribution of number and location of painful sites among FF, (2) to determine whether work limitations differ among FFs based on the number or location of painful sites, (3) to assess whether age and or sex influenced work limitation among FFs, (4) to determine whether the number of self-reported non-MSK comorbid health condition predicts work limitations among FFs, (5) to assess if age and years of service influences work limitations among FFs with non-MSK comorbid conditions.

## 2. Materials

### 2.1. Study Design/Setting

This was a cross-sectional study that utilized convenience sampling. Participants included men and women professional/career FFs between the ages of 18 and 60 years, recruited primarily from the city of Hamilton, Ontario, for the Firefighter Injury Reduction Enterprise: Wellness Enabled Life & Livelihood (FIREWELL) study between January 2013 and December 2014. Due to the underrepresentation of women FFs in the city of Hamilton Ontario, more women FFs were recruited from other cities across Canada. The recruitment strategies for this study involved online and in-person administration of the surveys. Firefighters completed the questionnaires in two formats: paper-based (distributed at conference Calgary, state of Alberta) and web-based (via an open-source survey tool, LimeSurvey—Hamburg, Germany) as emails were sent out to firefighter stations.

### 2.2. Ethics Approval and Consent to Participate

Ethical approval was received from the McMaster Research Ethics Board (#:14-540). Written and signed voluntary informed consent was obtained from all participants before the commencement of the study.

### 2.3. Study Participants (Inclusion Criteria)

The study participants include consenting men and women FFs between the ages of 18 and 60 years in fire service.

### 2.4. Variables

FFs completed several self-report measures, including a work limitation questionnaire and a questionnaire for sociodemographic factors (years in firefighting service), and self-reported anthropometry (age, sex, height, and weight). Participants had a choice to complete either a web-based questionnaire administered via a lime survey tool or a paper-based questionnaire administered by research staff. Responses for the presence of pain were answered as either a “yes” or “no,” and the location of body pain was indicated based on a checklist of “location of body” provided in the questionnaire. These locations of the body included the following: head, neck, shoulder, arm/elbow/hand, back, stomach/abdomen, upper thigh, knee, lower leg, foot, and others.

### 2.5. Data Sources/Measurement

#### 2.5.1. Health Problem

The Katz self-administered comorbidity questionnaire was used to assess the presence of a comorbid health condition among FFs. Responses for the presence, treatment, and limitations by the comorbid conditions were answered as either “yes” or “no.” This was used to categorize non-MSK health conditions (heart disease, high blood pressure, diabetes, cancer, depression, lung disease, ulcer or stomach disease, kidney disease, anemia or other blood problems, and other medical diseases) as follows: 0 = having no health problems, 1 = having one health problem, and 2 = having two or more health problems.

#### 2.5.2. The Work Limitations Questionnaire (WLQ-26)

The work limitations questionnaire (WLQ-26) is derived from the WLQ-25 that was initially developed and tested in persons with chronic conditions [10]. The WLQ-26 consists of 26 items which are divided into 4 subscales: time, mental-interpersonal, output, and physical work limitations [10]. The physical limitations subscale asks questions concerning the participants' ability to carry out tasks that involve muscle strength, endurance, and coordination. The mental-interpersonal limitations subscale questions the participants' ability to carry out cognitive tasks and social interactions at work. Finally, the output limitations ask questions that cover productivity on the job while time limitations address difficulty handling timeliness and scheduling demands at work [10]. The WLQ-26 has been utilized in various working populations with musculoskeletal injuries or other chronic conditions [17]. It takes less than 10 minutes to complete, and each subscale is scored on a Likert scale ranging from 0 to 4 (0 = difficulty none of the time, 1 = difficulty a bit of the time, 2 = difficulty at some of the time, 3 = difficulty most of the time, 4 = difficulty all of the time) [10]. A total score ranges between 0 (no limitations) and 100 (most limitations). The WLQ-26 has been found to have good construct validity and content validity [15]. It is sensitive to change with a standardized responsive mean of 0.65 and a minimally clinically important difference (MCID) of 13/100 points for summed score [10].

#### 2.5.3. Study Size

We did not perform a sample size calculation.

### 2.6. Statistical Methods

All statistical analysis was conducted using the STATA/14.2C software. The scores of individual items of the work limitations score were summed, averaged, and standardized to a range of 0–100, with a higher score indicating more limitations. Visual impression using a histogram of the total score of the work limitation data and each of the subscale showed that the data were skewed to the left. Requisite assumptions were also tested using the Shapiro-Wilk test of normality (alpha ≤ 0.05), confirming the data were skewed with an unequal variance for the number or location of painful sites. Therefore, a stabilizing transformation (square root of the work limitations score) was utilized to normalize the data to meet the requisite assumptions. Histograms and the Shapiro-Wilk test demonstrated that the transformed scores (0–10) were normally distributed. Descriptive summary statistics were calculated as the median and interquartile range for the untransformed variables of interest including time, physical, output, and mental-interpersonal work limitations scores. The means and standard deviations were utilized for demographic characteristics while frequencies and percentages were derived for the location or number of painful sites.

The chi-squared analysis was used to test the proportion between demographic factors (sex, age, BMI, and years in the fire service) and the location or number of body pain of FFs. Furthermore, age was categorized as being >45 and ≤45 years and years of fire service as 0 to 10 years, >10 to 20 years, and >20years. A one-way ANOVA was employed to analyze the transformed work limitations scores; this was a two-sided test with a significance level of <0.05. Painful locations were classified as follows: having no location of pain, upper extremity (shoulder, arm, elbow, and hand), lower extremity (upper thigh, knee, foot, and lower leg), and spine (back, head, and neck). The number of painful sites was also divided into having no pain, one location of pain, two locations of pain, and three or more locations of pain. Each transformed subscale of physical, time, output, and mental work limitations was treated as a dependent variable while the number of painful sites and the location of pain were the independent variables. When the overall effects were identified, a post hoc test was conducted to further determine where the differences existed generally for FFs, and for both men and women FFs. Univariate linear regression models were constructed with work limitations (time, output, mental, and physical subscales) as a dependent variable and nonmusculoskeletal comorbid health conditions (0, 1, 2, or more comorbidities) as a predictor—independent variable. Multivariate models using backward elimination were constructed using each subscale as a dependent variable while the age, years in fire service, and non-MSK comorbid conditions were predictors. Also, separate univariate and multivariate models were run for men and women FFs as per sex and gender equity research (SAGER) guidelines.

## 3. Results

### 3.1. Participants and Descriptive Data

The data analyzed consisted of 325 (men = 216, women = 109) FFs in total.  Table 1 shows the participant characteristics. Men FFs had a mean (SD) age of 42.6 (±8.7) years while women FFs had a mean age of 35 (±8.5) years. The chi-square analysis showed that the BMI categories among men and women FFs were significant (*χ*^2^_(3)_ = 55.8; *p* < 0.05) as men FFs had higher BMI than women FFs. The years in the fire service among men and women FFs was also significant (*χ*^2^_(2)_ = 63.9; *p* < 0.05). There was an equal distribution of men and women FFs between 0 and 10 years in the fire service. However, a larger proportion of men FFs are represented in the fire service between >10 to 20 years (76.4% vs. 23.5%; *p* < 0.05) and >20 years (94.3% vs. 5.7%; *p* < 0.05) than women FFs.

### 3.2. Number and Location of Body Pain

**Figure 1:**
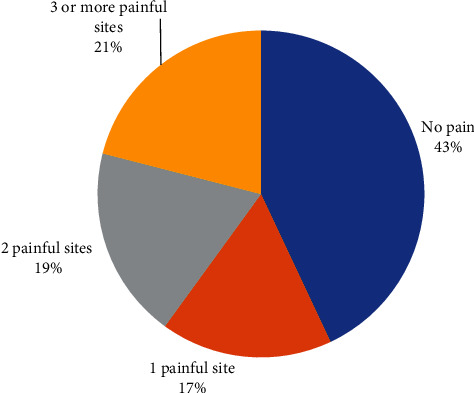
Number of painful sites.

**Figure 2:**
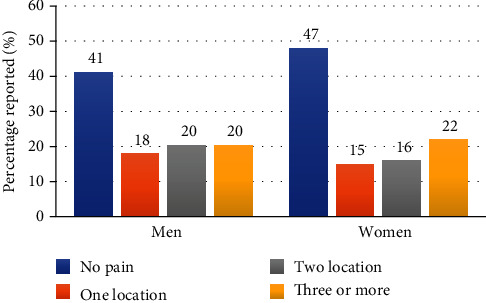
Number of painful sites by sex.

**Figure 3:**
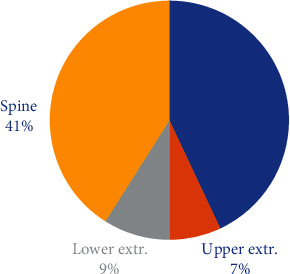
Location of painful sites.

**Figure 4:**
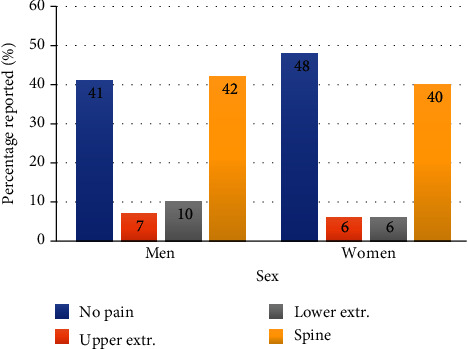
Location of painful sites of sex.

Figure 1 displays the percentage distribution of the number of painful sites in our study cohort: 43%—no pain, 17%—one painful site, 19%—two painful sites, and 21%—three or more painful sites.  Figure 2 displays these distributions by sex. Among the men subgroup of 216 firefighters, 41.2%—no pain, 18%—one painful site, 20.4%—two painful sites, and 20.4%—three or more painful sites. Among the woman subgroup of 109 firefighters, 47%—no pain, 15%—one painful site, 16%—two painful sites, and 22%—three or more painful sites.  Figure 3 reports the percentage distribution of the locations of painful sites in our study cohort: 43%—no pain, 41%—spine, 9%—lower extremity, and 7%—upper extremity.  Figure 4 displays these distributions by sex. Among the men subgroup of 216 firefighters, 41%—no pain, 42%—spine, 10%—lower extremity, and 7%—upper extremity. Among the women subgroup of 109 firefighters, 48%—no pain, 40%—spine, 6%—lower extremity, and 6%—upper extremity.

### 3.3. Work Limitation Scores

The median work limitation scores range from 3.1/100 to 15.6/100 for the number of painful sites and 0/100 to 17.7/100 for the location of body pain. The untransformed median work limitation scores for each WLQ-26 subscale are displayed in Table 2. There was no significant effect between the transformed average work limitation scores of FFs and other covariates including sex, BMI, and years in the fire service. However, the difference between the transformed average work limitation score and age categories had a significant effect in FFs (*F*_1,314_ = 7.11, *p* = 0.008). FFs > 45 years of age experienced more physical work limitations than FFs ≤ 45years (mean difference: 0.74/10; 95% CI .19-1.29; *p* = 0.008). The median and interquartile range work limitation scores (/100) of each subscale are displayed in Table 3.

### 3.4. Non-MSK Comorbidity by Demographic Characteristics

**Figure 5:**
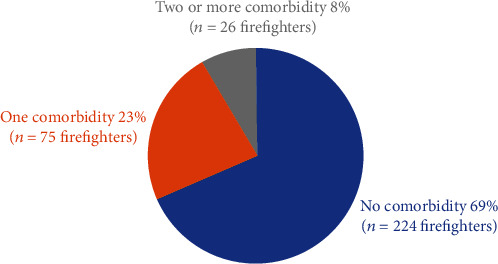
Number of comorbidities.

**Figure 6:**
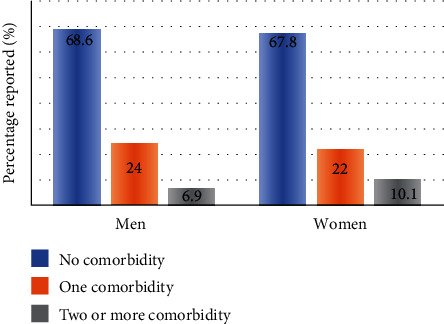
Number of comorbidities by sex.

An estimated 31% of FFs (*n* = 102) reported non-MSK comorbidity with 23% (*n* = 76) reporting at least one non-MSK comorbidity and 8% (*n* = 26) reported having two or more comorbidities. See Figures 5 and 6. The most reported type of non-MSK comorbidity among FFs was having depression 9.2% (*n* = 30) and high blood pressure 8.6% (*n* = 28) Table 4.

### 3.5. Effects of Number of Painful Sites and Location of Body Pain on Work Limitation

The mean differences and confidence interval pertaining to physical, mental, output, and time limitations are displayed in Table 5.

*Physical limitation*: the number of painful sites and location of body pain showed significant differences in physical work limitations (*p* < 0.05). *Number of painful sites—*FFs with three or more painful sites (mean difference = 1.0/10; 95% CI: 0.1–1.9; *p* = 0.02) experienced more physical limitations compared to FFs with no painful sites. There was no significant effect of physical limitation based on the location of pain among women FFs. *Location of body pain—*FFs with spinal pain (mean difference = 0.8/10; 95% CI: 0.1–1.6; *p* = 0.01) experienced more physical limitations compared to FFs with no pain. In addition, there was a significant effect of physical limitation (*F*_3,207_ = 3.01, *p* = 0.03) between men FFs who reported spinal pain (*p* = 0.02) and men FFs without pain.

*Mental limitations*: the number of painful sites and location of body pain showed significant differences in mental work limitations (*p* < 0.05). *Number of painful sites—*FFs with two painful sites (mean difference 1.1/10; 95% C.I: 0.3–2.0; *p* = 0.004) and three or more painful sites (mean difference = 1.2/10; 95% CI: 0.3–2.0; *p* = 0.002) experienced more mental limitations compared to FFs with no painful sites. Men FFs with two, or three or more painful locations reported a significant effect for mental limitation than men FFs without pain. Women FFs with three or more painful sites reported a significant effect (*p* = 0.02) and experienced more mental work limitations than women FFs without pain. *Location of body pain—*FFs with upper extremity pain (mean difference = 1.4/10; 95% CI: 0.1-2.7; *p* = 0.03) and spinal pain (mean difference = 0.9/10; 95% CI: 0.2-1.6; *p* = 0.003) experienced more mental limitations compared to FFs with no pain. There was a significant effect of mental limitation (*p* = 0.02) between men FFs who reported spinal pain (*p* = 0.02) and those without pain. In addition, there was a significant effect of mental limitation (*p* = 0.01) between women FFs with upper extremity pain (*p* = 0.05) and women FFs without pain.

*Output limitations*: the location of body pain only showed significant differences for output limitations (*p* = 0.01). *Location of body pain—*FFs with spinal pain (mean difference = 0.8/10; 95% CI: 0.1–1.6; *p* = 0.02) experienced more output limitations compared to FFs with no pain. There was no significant effect of output limitations based on the location of pain among men FFs. By sex, there was a significant difference in the output limitation (*p* = 0.01), as women FFs with lower extremity pain (*p* = 0.02) experienced more output limitations than women FFs without pain.

*Time limitations*: there was no significant difference between FFs' number of painful sites or location of body pain on time limitation when compared to FFs with no pain. Furthermore, there was no significant difference among men and women FFs' location of painful sites on the time limitation.

### 3.6. Work Limitations by the Number of Non-MSK Comorbidity

Overall, in predicting work limitations among firefighters (Table 6), the number of comorbidities had minimal predictive value (physical *R*^2^ = 0.01, mental *R*^2^ = 0.06, output *R*^2^ = 0.04, time *R*^2^ = 0.02). Furthermore, in our multivariate regression model, predicting work limitations among firefighters (Table 7), the number of comorbidities, age, and years of service had minimal predictive values (physical *R*^2^ = 0.01, mental *R*^2^ = 0.06, output *R*^2^ = 0.04, time *R*^2^ = 0.02).

## 4. Discussion

### 4.1. Key Results

Our findings indicated that nearly six in ten firefighters (57%) were reported as having at least one painful site in our study cohort. Approximately four in ten firefighters (42%) indicated the spine as the most common painful location. Nearly, 1 in 3 firefighters was reported as having at least one non-MSK comorbid conditions (31%), with an almost uniform prevalence in women FFs (32%) and men FFs (31%). Our study also showed generally low median work limitation scores among FFs despite having at least one non-MSK comorbidity. In addition, there was a small but significant impact of non-MSK comorbidity on work limitations among FFs. Age also had an impact on FFs' physical work limitations—FFs aged >45 years experienced more physical work limitations than those ≤45 years of age. Furthermore, ≥3 painful sites and spine and upper extremity pain may potentially contribute to physical/mental and work output limitations.

A large proportion of our cohort indicated having at least one painful site. When stratified by sex, these proportions did not vary greatly—58.8% of men FFs and 53% of women FFs were reported as having at least painful sites. Our results were in agreement with the Carleton et al. and Nazari et al. (2019) studies of a high prevalence of pain among firefighters [3, 18]. Carleton et al. indicated that the prevalence of chronic pain in a sample of 807 firefighters was 35% [18]. Similarly, in the Nazari et al. 2019 study, the prevalence estimates of 17–27% were reported for the neck, shoulder, arm/elbow/hand, back, and knee regions [3]. The reported proportion of at least one painful site (57%) was 3 times higher than that of the prevalence of chronic pain among the Canadian general population (19%) [19]. Although these proportions cannot entirely and statistically be compared to the general population estimates in 2007-2008, they do appear higher. The spine (back) region was considered the most commonly reported painful location in our cohort. When stratified by sex, these proportions were nearly similar—42% of men FFs and 40% of women FFs reported the spine as the most common painful location. Both the Carleton et al. and Nazari et al. (2019) studies also indicated the spine (back) as the most commonly painful anatomical region with prevalence estimates of 18% (in a sample of 807 firefighters) and 27% (in a sample of 1491 firefighters), respectively [3, 18].

Age also had an effect on FFs' physical work limitations. In our cohort of 325 firefighters (216 men; 109 women), FFs aged >45 years experienced more physical work limitations than those ≤45 years of age. These findings were in keeping with previous studies that highlighted age as an important variable that affects injury and task performance in firefighters. Sinden et al. displayed that the performance of firefighting tasks such as the hose drag was adversely influenced by increased age in the fire service [20]. Higher cardiorespiratory fitness levels are associated with better firefighting task performance [1, 2]. The Nazari et al. (2017) study indicated that cardiorespiratory fitness levels declined with aging among firefighters [2]. Further, the decline rates in cardiorespiratory fitness levels were similar among both firefighters and healthy participants [2]. However, it is important to note that it is difficult to distinguish between age-related changes from cumulative strains and effects of repetitive overuse injuries on musculoskeletal system [8].

Our study also identified that FFs with multiple painful sites experienced more physical and mental limitations compared to FFs with no painful sites. This is due to the fact that pain is a multifaceted disorder and truly a biopsychosocial experience with physical and mental health contributions and sequelae and is associated with substantial disability and burden to the population, health care systems, and societies [19]. Firefighter-specific occupational experiences and circumstances and work-related injuries are also likely contributing factors to our findings [1, 2, 8, 20]. Beyond the physical limitations, firefighters are also frequently exposed to potentially traumatic events [18]. There is a significant link between mental disorders (posttraumatic stress disorder in particular) and chronic pain in firefighters and other public safety personnel [18]. Therefore, the high proportion (57%) of our firefighter cohort who indicated having at least one painful site may be closely linked to the potentially traumatic nature of their occupation. This was evident in our study as the results indicated that men FFs with spinal pain and women FFs with upper extremity pain experienced more mental limitations.

The prevalence of non-MSK comorbidities was 31% among FFs with an almost uniform prevalence in women FFs (32%) and men FFs (31%). Plat et al. [21] examined the impact of comorbidities among Dutch FFs. The result showed that about a quarter (23%) of FFs reported the presence of at least a comorbidity; however, the comorbidity did not impact the work ability of the FFs. The difference in the result of both studies might be related to the different geographic locations under study. Women FFs are often excluded from studies, but our study had a significant sample of women FFs. Hence, our findings showed that women FFs having at least one non-MSK comorbid health condition experience greater physical limitations. Older age showed a small but significant association with greater output and mental work limitations in this study. This concurred with a study by Slater et al. [22], who reported that comorbid health condition increases the risk of physical limitations specifically in persons with existing comorbidities.

### 4.2. Strengths and Limitations

The current study strengths include using a large sample of firefighters from Hamilton, Ontario, with a good representation of both sexes, and therefore, to a certain extent, can be considered representative of a larger population. Most FF studies usually exclude the women FFs because they occupy a small percentage of the entire FF population. Our study presented unique data on a large sample of women FFs. The study was a cross-sectional study; hence, it does not provide a definitive information about the cause and effect relationship between the location of the body or the number of pain sites in the body and work limitations among FFs. A convenience sample along with the use of two different data collection strategies (online and paper) cannot be considered a representative sample of the general population. We were unable to provide specific diagnoses or the type of health problems that might have originally caused the pain at a given location reported by FFs. We also found generally low levels of work limitations, but given the high demand tasks that FFs perform, the WLQ-26 may not adequately represent the highly demanding tasks of FFs. This is not surprising as FFs are more likely to exhibit the healthy worker effect due to a lower morbidity and mortality rate at work compared to the general population. Therefore, a self-report performance limitation scale designed for FFs may be needed to identify the limitations at work.

### 4.3. Implications for Future Research

This work indicates the important link between the painful locations and both physical and mental demands and limitations among firefighters at work. Although firefighting is thought of as a physically demanding job which can cause pain and exposure to a high risk of traumatic events, the overlap between physical and mental health may be underappreciated. Future studies should explore this interrelationship, with trauma being a common pathway for both musculoskeletal and mental health problems. This finding further highlights the importance of developing firefighter-specific injury prevention, rehabilitation, and mental wellness programs.

## 5. Conclusion

Approximately six in ten firefighters (57%) were reported as having at least one painful site, and nearly four in ten firefighters (42%) indicated the spine as the most common painful location in our study cohort. Approximately, 1 in 3 firefighters was indicated as having at least one non-MSK comorbid conditions (31%). Further, a generally low median work limitation score among FFs despite having at least one non-MSK comorbidity was reported. FFs aged >45 years experienced more physical work limitations than those ≤45 years of age. Additionally, we identified that reporting of ≥3 painful sites, spine and upper extremity pain, may potentially contribute to the physical/mental and work output limitations. The number of comorbidities, age, and years of service had minimal value in predicting work limitations among FFs.

## Figures and Tables

**Table 1:** Participants demographics.

Demographics characteristics	All	Men	Women
Number of participants (%)	325	216 (66%)	109 (34%)
	Mean (SD)		
Age (yrs.)	39.9 ± 9.4	42.6 ± 8.7	34.7 ± 8.5
Height (m)	1.8 ± 0.3	1.8 ± 0.3	1.7 ± 0.1
Weight (kg)	83.2 ± 14.2	89.9 ± 11.3	70.5 ± 9.5
BMI (kg/m^2^)	26.8 ± 4.3	27.8 ± 4.1	24.7 ± 4.0
Years of service (yrs.)	12.9 ± 8.6	15.9 ± 8.1	7.4 ± 7.5

**Table 2:** Median and interquartile range (IQR) work limitation scores for the number or body location of painful sites.

Work limitations scores	Number of painful sites			Body location		
	One painful site	Two painful sites	Three or more painful sites	Upper extremity	Lower extremity	Spine
Physical limitations	3.1 (0, 12.5)	3.1 (0, 15.6)	6.3 (0, 15.6)	3.1 (0, 15.6)	0 (0, 15.6)	6.3 (0, 12.5)
Output limitations	12.5 (6.2, 25)	12.5 (6.2, 25)	12.5 (6.2, 18.7)	12.5 (6.2, 25)	12.5 (3.1, 21.8)	12.5 (6.2, 18.7)
Time limitations	8.3 (0, 16.6)	8.3 (4.1, 16.6)	8.3 (4.1, 16.6)	12.5 (6.2, 25)	6.2 (0, 16.6)	8.3 (4.1, 16.6)
Mental limitations	15.6 (3.1, 21.8)	15.6 (6.2, 25)	15.6 (6.2, 25)	17.1 (9.3, 31.2)	12.5 (3.1, 21.8)	15.6 (6.2, 25)

Range of work limitation scores for each subscale = 0 − 100. Higher scores denote greater work limitations.

**Table 3:** Median and interquartile range (IQR) work limitation scores.

Work limitations scores	No comorbidity Median (IQR)	One comorbidity Median (IQR)	Two or more comorbidity Median (IQR)
Physical limitation scores	0 (0, 9.3)	1.6 (0, 12.5)	0 (0, 12.5)
Mental limitation scores	12.5 (3, 21.8)	15.5 (6.2, 28.1)	12.5 (0, 18.7)
Time limitation scores	4.2 (0, 16.6)	8.3 (0, 16.6)	4.2 (0, 12.5)
Output limitation scores	6.3 (0, 18.7)	12.5 (6.2, 25)	6.3 (0, 18.7)

**Table 4:** Prevalence of the type of non-MSK comorbidity among FFs.

Frequency (%)	All	Male	Female
Presence of comorbidity	102 (31.4%)	67 (31%)	35 (32%)
Heart disease	6 (1.8%)	5 (2.3%)	1 (0.9%)
High blood pressure	28 (8.6%)	21(9.7%)	7 (6.5%)
Lung disease	4 (1.2%)	3 (1.4%)	1 (0.9%)
Diabetes	2 (0.6%)	2 (0.9%)	0
Ulcer/stomach pain	6 (1.8%)	5 (2.3%)	1 (0.9%)
Kidney disease	0	0	0
Anaemia	11 (3.3%)	6 (2.8%)	5 (4.6%)
Cancer	5 (1.5%)	4 (1.8%)	1 (0.9%)
Depression	30 (9.2%)	17 (7.7%)	13 (11.7%)
Others	41 (12.5%)	27 (12.4%)	14 (12.8%)

**Table 5:** Effect of area of body pain and number of painful sites on work limitation in FFs.

Work limitation scores	Number of painful sites			Body location		
	One painful site	Two painful sites	Three or more painful sites	Upper extremity	Lower	Spine
Physical limitations	0.6 (-0.3-1.6)	0.7 (-0.2-1.7)	1.0^∗^ (0.1-1.9)	0.8 (-0.6-2.2)	0.4 (-0.8-1.7)	0.8^∗^ (0.1-1.6)
Mental limitations	0.5 (-0.4-1.5)	1.1^∗^ (0.3-2.0)	1.2^∗^ (0.3-2.0)	1.4^∗^ (0.9-2.7)	0.9 (-0.3-2.0)	0.9^∗^ (0.2-1.6)
Time limitations	0.6 (-0.4-1.5)	0.7 (-0.2-1.6)	0.7 (-0.2-1.5)	1.3 (-0.1-2.6)	0.5 (-0.7-1.7)	0.6 (-0.1-1.3)
Output limitation	0.9 (-0.03-1.9)	0.8 (-0.1-1.8)	0.8 (-0.1-1.7)	1.0 (-0.4-2.5)	0.9 (-0.4-2.1)	0.8^∗^ (0.1-1.6)

Reference group: having no region or number of painful sites. ^∗^Significant at *p* < 0.05.

**Table 6:** Univariate regression results for the work limitation sub-scales among firefighters.

Overall	Physical (*R*^2^ = 0.01)		Mental (*R*^2^ = 0.06)		Output (*R*^2^ = 0.04)		Time (*R*^2^ = 0.02)	
	*β* (S.E)	*ρ*	*β* (S.E)	*ρ*	*β* (S.E)	*ρ*	*β* (S.E)	*ρ*
One comorbidity	2.39 (1.76)	0.17	4.48 (2.06)	0.03	2.84 (2.04)	0.16	1.51 (1.85)	0.41
Two or more CM	-1.53 (2.77)	0.58	-2.26 (3.21)	0.48	-2.64 (3.18)	0.40	1.13 (2.95)	0.70
Constant	7.27(.89)	<0.05	14.76 (1.05)	<0.05	12.64 (1.03)	<0.05	10.09(.93)	<0.05
Male	Physical (*R*^2^ = 0.004)		Mental (*R*^2^ = 0.02)		Output (*R*^2^ = 0.01)		Time (*R*^2^ = 0.001)	
	*β* (S.E)	*ρ*	*β* (S.E)	*ρ*	*β* (S.E)	*ρ*	*β* (S.E)	*ρ*
One comorbidity	0.66 (2.32)	0.77	3.57 (2.58)	0.16	2.33 (2.56)	0.36	.13 (2.24)	0.95
Two or more CM	-3.48 (4.03)	0.38	-5.20 (4.35)	0.23	-4.88 (4.30)	0.25	-1.77 (3.67)	0.62
Constant	8.16 (1.19)	<0.05	15.41 (1.32)	<0.05	12.80 (1.32)	<0.05	10.66 (1.13)	<0.05
Female	Physical (*R*^2^ = 0.07)		Mental (*R*^2^ = 0.04)		Output (*R*^2^ = 0.01)		Time (*R*^2^ = 0.04)	
	*β* (S.E)	*ρ*	*β* (S.E)	*ρ*	*β* (S.E)	*ρ*	*β* (S.E)	*ρ*
One comorbidity	6.27 (2.40)	0.01	6.71 (3.45)	0.05	4.05 (3.36)	0.23	4.64 (3.31)	0.16
Two or more CM	1.72 (3.18)	0.58	2.23 (4.66)	0.63	0.80 (4.56)	0.86	6.77 (4.94)	0.17
Constant	-2.01 (7.21)	<0.05	13.39 (1.72)	<.05	12.31 (1.62)	<0.05	8.84 (1.63)	<0.05

**Table 7:** Multivariate regression results for the work limitation subscales among firefighters.

Overall	Physical (*R*^2^ = 0.01)		Mental (*R*^2^ = 0.06)		Output (*R*^2^ = 0.04)		Time (*R*^2^ = 0.02)	
	*β* (S.E)	*ρ*	*β*(S.E)	*ρ*	*β* (S.E)	*ρ*	*β* (S.E)	*ρ*
One comorbidity	-	-	4.25 (2.06)	0.04^∗^	-	-	-	-
Two or more CM	-	-	.75 (3.20)	0.81	-	-	-	-
Age	.31 (.07)	<0.05	0.28 (.09)	0.04	0.27 (.09)	0.004	0.17 (.08)	0.04
Years of service	-	-	-.25(.10)	0.02^∗^	-.22 (.10)	0.03^∗^	-	-
Constant	-4.38 (3.21)	0.12	14.76 (1.05)	<0.05	5.05 (3.77)	0.18	3.49 (3.50)	0.31
Male	Physical (*R*^2^ = 0.004)		Mental (*R*^2^ = 0.02)		Output (*R*^2^ = 0.01)		Time (*R*^2^ = 0.001)	
	*β* (S.E)	*ρ*	*β* (S.E)	*ρ*	*β* (S.E)	*ρ*	*β* (S.E)	*ρ*
Age	.39 (.11)	0.001	0.20 (.12)	0.09	0.19 (.12)	0.12	-	-
Constant	-8.50 (4.89)	0.08	6.99 (5.47)	0.20	4.86 (5.44)	0.37	-	-
Female	Physical (*R*^2^ = 0.07)		Mental (*R*^2^ = 0.04)		Output (*R*^2^ = 0.01)		Time (*R*^2^ = 0.04)	
	*β* (S.E)	*ρ*	*β* (S.E)	*ρ*	*β* (S.E)	*ρ*	*β* (S.E)	*ρ*
Age	.11(.12)	0.35	0.40(.16)	0.02	.43(.16)	0.01	-	-
Constant	-1.43 (4.39)	0.74	0.95 (5.94)	0.87	-1.63 (5.83)	0.78	-	-

## Data Availability

The data cannot be made available to readers upon request as we, the authors, do not have ethical approval to share the data.
